# Impact of Snoring on the Cardiovascular System and its Treatment: Positive and Negative Effects of Continuous Positive Airway Pressure in Sleep Apnea

**DOI:** 10.7759/cureus.9796

**Published:** 2020-08-17

**Authors:** Thulasi Priya Jayaprakash, Olisaemeka D Ogbue, Parul Malhotra, Radhika Akku, Safeera Khan

**Affiliations:** 1 Internal Medicine, California Institute of Behavioral Neurosciences and Psychology, Fairfield, USA; 2 Medicine, California Institute of Behavioral Neurosciences and Psychology, Fairfield, USA; 3 Internal Medicine, Punjab Institute of Medical Sciences, Ludhiana, IND

**Keywords:** sleep apnea, cardiomyocytes, cpap

## Abstract

Obstructive sleep apnea (OSA) is a common condition, and if not treated can be a significant risk factor for multiple comorbidities like hypertension (HTN), coronary artery disease (CAD), and congestive heart failure (CHF). The underlying pathophysiology involves coagulation and inflammatory pathways, including an overactive sympathetic nervous system. This ultimately causes hemodynamic changes and subclinical myocardial injuries. We reviewed the published literature about the impact of continuous positive airway pressure (CPAP) when used as a mode of treatment to reduce the OSA effects on cardiomyocytes. We found that the results were mixed, including both ill and good effects. The cardiac markers like N-terminal pro-brain natriuretic peptide (NT-proBNP) and atrial natriuretic peptide (ANP) were reduced, implying the decrease in the incidence of heart failure with CPAP treatment in a few of the studies. They also proved a significant decrease in harmful cardiovascular (CV) outcomes, while others concluded that CPAP therapy might be stressful on the heart, causing an elevation in cardiac troponin T levels. However, the impact on inflammatory markers is still indeterminate and needs more research in future.

## Introduction and background

Obstructive sleep apnea (OSA) occurs when the soft tissue muscles of the oral cavity like tongue and soft palate relax temporarily and fall back causing narrowing of the airway. Untreated OSA was found to be a significant risk factor for many comorbidities like arterial hypertension (HTN), congestive heart failure (CHF), coronary artery disease (CAD), myocardial ischemia (MI), atrial fibrillation (AF), and ventricular tachycardia. Coagulation pathway and inflammatory pathway abnormalities, overactive sympathetic system, vascular endothelial dysfunction, and metabolic dysregulation are most likely the underlying pathophysiological mechanisms involved [1]. OSA leads to hemodynamic changes that cause an increase in preload and afterload [2]. These changes further lead to the development of hypoxemia during sleep and, thereby, an increase in oxygen demand. The outcome is a subclinical myocardial injury [2]. There is considerable evidence available that links an association between obstructive sleep apnea-hypopnea syndrome (OSAHS) and increased cardiovascular (CV) morbidity and mortality. However, the involved pathological mechanism is not clear, nor is the role of cardiac biomarkers [3]. According to The Sleep Heart Health Study, Wisconsin Sleep Cohort Study, and the Seventh Report of the Joint National Committee on the management of high blood pressure, OSA is one of the major identifiable causes of HTN [4].

Recent studies have established a relationship between OSA and the following key biomarkers: a) endothelin-1 (ET-1), a marker of endothelial dysfunction; b) high sensitivity troponin T (hs Trop-T), a marker of myocardial injury; c) high-sensitivity C-reactive protein (hs-CRP), a marker of inflammation; d) cardiac neurohormone N-terminal pro-brain natriuretic peptide (NT-proBNP), a marker of ventricular strain, and e) fibrinogen, a marker of hypercoagulation [5]. Several randomized controlled trials have evaluated the effect of continuous positive airway pressure (CPAP) on these biomarkers. Researchers are trying to prove the benefits of CPAP as first-line treatment in OSA in reducing daytime sleepiness and improving the quality of life [6]. There is sufficient data available to prove the decrease in the incidence of premature ventricular beats, brain natriuretic peptide (BNP) levels in CHF patients, new CV events in CAD, and HTN in coronary artery bypass graft patients [1,3,5]. Although the CPAP’s usefulness on hemodynamic parameters like blood pressure, preload, afterload, and cardiomyocyte function is consistent according to some observers [5], others state that the association of OSA severity and the biomarkers, as well as the effects of CPAP on circulating levels of these biomarkers, is still to be established conclusively [7]. In this literature review, we are trying to analyze whether CPAP therapy in mild, moderate, and severe OSA patients has a beneficial effect on cardiomyocyte function or not. Moreover, CPAP treatment guidelines are yet to be established [4].

## Review

**Figure 1:**
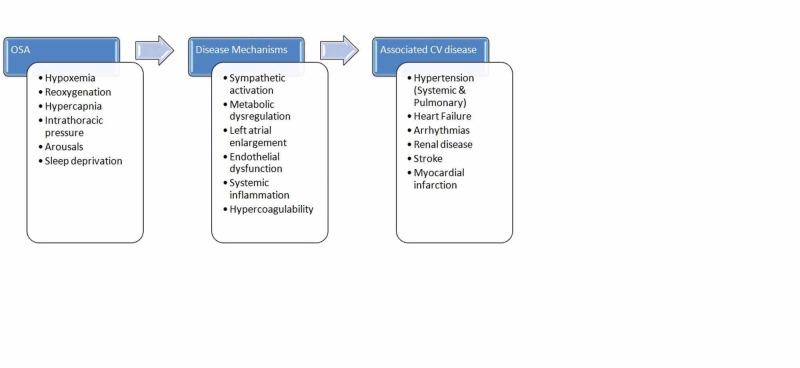
A depiction of the pathophysiology of associated cardiovascular disease in obstructive sleep apnea

Multiple underlying pathophysiological mechanisms are involved in the development of various CV problems, like systemic or pulmonary HTN, heart failure (HF), and renal disease in OSA patients. A detailed picture illustrating the connections between these pathological processes and the resultant outcome is shown below in Figure 1.

Cardiac biomarkers

The impact of CPAP on different cardiac biomarkers is variable. The natriuretic peptides like atrial natriuretic peptide (ANP) and BNP levels are elevated in cardiac failure due to a stretch on the ventricular walls. An observational study done in 2008 by Kita et al. showed a reduction in natriuretic peptide levels with nasal CPAP usage overnight [8]. But the hemodynamic changes leading to an attenuated increase in blood pressure was more related to BNP than ANP [8]. While the decrease in BNP levels is beneficial, a negative influence of CPAP is also found in the form of elevated cardiac troponin levels. An observational study was done on CPAP effect on highly-sensitive troponin T plasma levels by Barceló et al. in 2014 [9]. The result was elevated cardiac troponin T (cTnT) due to the deleterious effect of CPAP, causing a potential degree of cardiac stress [9].

When the effect of CPAP was observed for a more extended period, changes in blood pressure and ventricular wall remodeling were noticed. Colish et al. conducted a study on cardiac remodeling by assessing cardiac biomarkers, echocardiography, and cardiac MRI at intervals of three months, six months, and 12 months [10]. It proved the reversal of both systolic and diastolic abnormalities and CV remodeling improvement by using CPAP therapy [10]. Some studies highlighted the importance of extended use of CPAP to observe the CV effects. However, even a single night application of positive pressure influenced cardiac biomarker levels in the blood. A study by Valo et al. in the year 2015, showed a significant decrease in NT-proBNP with a single night CPAP treatment; however, hs trop-T remained unaltered [2]. Two studies conducted in 2016 by Strehmel et al. and Msaad et al. showed a marked reduction in BNP levels in both normotensive and hypertensive patients; however, no significant changes were observed in cTnT levels due to the CPAP treatment [11,3].

Chang et al. conducted a randomized controlled trial whose results were similar to the observational studies mentioned above [12]. CPAP therapy was found to have a good reduction in NT-proBNP levels compared to their baseline levels in moderate to severe OSA patients. However, this study couldn't confirm CPAP therapy's effect on hs trop-T [12]. Efficacy of three months of CPAP treatment on high-sensitive troponin T (hs-TnT) in OSA patients without cardiovascular disease (CVD) was tested by a pilot study done by Zhang et al. in 2018, which demonstrated no significant differences [13]. The following table (Table 1) summarizes the studies that observed the effects of CPAP on cardiac biomarkers.

**Table 1:** Changes in cardiac biomarkers with CPAP therapy CPAP: continuous positive airway pressure; BNP: brain natriuretic peptide; hs TropT: high sensitivity troponin; OSA: obstructive sleep apnea; NT-proBNP: N-Terminal pro-brain natriuretic peptide; RCT: randomized clinical trial; TTE: transthoracic echocardiography; OSAHS: obstructive sleep apnea-hypopnea syndrome; ANP: atrial natriuretic peptide; CMR: cardiac MRI

Author/ Year	Purpose of the study	No. of patients	Type of study	Result/ Conclusion
Strehmel et al. [11]	Correlation of CPAP effectiveness with BNP and hs trop-T concentration in OSA and CAD	80	Observational	hs TropT: control=case, proBNP: control< case; no ST depression
Msaad et al. [3]	Changes in serum BNP in OSAHS patients with nasal CPAP	64	Observational	Significant decrease in BNP in both normotensive and hypertensive patients with CPAP treatment
Valo et al. [2]	MI markers in CAD and OSA patients and CPAP effects	21	Observational	Significantly reduced NT-proBNP with a single night CPAP ST depression attenuation unaltered hs trop-T
Chang et al. [12]	Effects of CPAP in OSA patients on trop T and BNP	37	RCT	Marked decrease in NT-proBNP and no significant reduction in hs trop-T with CPAP
Kita et al. [8]	Response of nighttime release of cardiac natriuretic peptides during OSA with nasal CPAP	22	Observational	BNP differs from ANP in secretion profile and hemodynamic changes are related to BNP during apnea in OSA patients
Colish et al. [10]	Effects of CPAP in OSA patients on cardiac remodelling by assessing cardiac biomarkers, echocardiography, and cardiac MRI	47	Observational	With CPAP therapy as early as three months, a reversal of both blood pressure abnormalities, and for one year gradual improvement in cardiovascular remodelling are noticed that are assessed with both TTE and CMR
Zhang et al. [13]	Efficacy of CPAP treatment on hs TnT in OSA patients without CV disease	93	Pilot study	With three months of CPAP therapy there was no significant change in hs trop-T
Barceló et al. [9]	Plasma hs trop-T levels in patients with OSA- an effect of CPAP treatment	200	Observational	A potential degree of cardiac stress which caused deleterious effects on the heart might be a result of CPAP treatment

Evidence Level

These studies show that the natriuretic peptides like ANP and BNP are markedly reduced irrespective of the duration of CPAP therapy. Thereby, a decrease in the incidence of HF is evident. However, there was not much effect on the myocardial ischemic biomarker, the highly sensitive troponin T (hs trop-T), which even increased at times. This, however, might be related to the cardiac stress that is associated with CPAP usage.

Cardiovascular outcomes

Increased myocardial oxygen consumption and decreased oxygen supply in OSA are the main reasons for the nocturnal ischemia. Peled et al. found that these ischemic events could be minimized by a combination of CPAP treatment and medical therapy to control heart rate and elevated blood pressure. In particular, they noticed significantly ameliorated nocturnal ST depression time with CPAP usage [14]. In an observational study conducted by Seyis et al., a considerable improvement was seen in pathological changes like a decrease in the frequency of premature ventricular contractions (PVCs), transmural dispersion of ventricular repolarisation (Tp-e), QTc dispersion, and Tp-e/QTc ratio, after six months of CPAP treatment, ultimately leading to improvement in cardiac function in patients with HF and OSA [1].

Another study by Kasai et al. highlighted the prognosis of HF in OSA patients treated with CPAP [15]. They concluded that CPAP usage reduced the incidence of hospitalization as well as mortality rates among those with HF and OSA, decreased compliance to CPAP treatment has shown higher morbidity and mortality rates [15]. The benefits of CPAP were also evident in post-coronary artery bypass graft (CABG) patients, along with HF and CAD. Dong et al. conducted an observational study on 59 hypertensive patients with CABG and OSA. They proved the importance of CPAP in a drop of systolic BP and improved non-dipping hypertensive status. Daytime somnolence was also alleviated [16].

Nocturnal blood pressure fluctuations (NBPF) are frequently seen in patients with CVD and OSA, which is, in turn, associated with the mean value of nocturnal BP and the arterial stiffness. Picard et al. did research and stated that CPAP treatment decreased the frequency of NBPFs, the mean value of the nocturnal BPs, and the arterial stiffness after six months of CPAP therapy [17]. It also improved all polysomnography derived parameters like mean apnea duration, arousal index, and sleep efficacy [17]. Marin et al. and Campos-Rodriguez et al. concluded in two different studies that adequate treatment with CPAP might reduce the CV death risks in men and women, respectively [18,19].

The overall incidence of CV deaths in sleep apnea patients reduced significantly with CPAP prescription regardless of the degree of apnea. Many meta-analyses showed an association between CPAP usage and a decrease in composite CV events, all-cause deaths, and CV deaths that were particularly demonstrated only in observational studies and not in randomized trials [20]. According to a systematic review and meta-analysis, done by Labarca et al., the data they collected from six randomized clinical trial (RCT)'s showed that they couldn't find any benefit from the usage of CPAP pertaining to improvement in major adverse cerebrovascular and cardiovascular endpoints (MACE) including unstable angina, MI, AF, and stroke along with all-cause mortality [21]. They also concluded that CPAP therapy was not beneficial in other CV related co-morbidities like type 2 diabetes mellitus, HTN, and dyslipidemia as well [21].

The outcome of CPAP usage resulting in a reduction in apneic events (especially obstructive sleep apnea) is more dependent on the compliance, the time duration, and the effective way of device application. Four multicenter RCTs with 3780 patients reviewed meta-analysis conducted by Abuzaid et al. in 2017 proved that CPAP decreased the incidence of MACE only in a particular subgroup, sleep apnea cardiovascular endpoints (SAVE) study, accounting for 65% of the meta-analysis subjects' sample who were wearing it for > four hours. The study also stated that the average CPAP usage was not sufficient to translate into MACE benefits [22]. However, it was discovered that CPAP is not effective in central sleep apnea (CSA) [22]. A meta-analysis by Haentjens et al. showed a good reduction in ambulatory mean BP with effective usage of CPAP at nights, which is reflected in terms of better prognosis in CV events [23]. Similar results were observed by Hu et al. [24]. Also, a substantial blood pressure reduction was noticed in patients who use antihypertensive therapy along with CPAP. Multiple factors like CPAP adherence, pressure, duration of usage, age, baseline systolic blood pressure (SBP), and OSA severity predict the outcome [24].

An absolute and relative increase in left ventricular ejection fraction (LVEF), improvement in daytime left ventricular (LV) systolic function in HF patients, in association with a decrease in LV end-systolic dimension, daytime SBP, and heart rate was detected in patients associated with sleep apnea [25]. The latest RCT conducted in 2017 by Glantz et al. showed non-reversal of worsened diastolic function in patients with CAD and heart failure with preserved ejection fraction (HFpEF) and non-sleepy OSA with CPAP prescription [26]. Although after adjusting all confounding factors and CPAP adherence for one year, their findings detected an improvement in diastolic relaxation velocity [26]. There was not much difference in CV events, with CPAP used as secondary prevention in patients who are already suffering from cardiac disease and sleep apnea. But there was a noticeable effect in decreasing snoring, daytime sleepiness, and improvement in the quality of life [27].

In 2010, a traditional review found that CPAP administration was beneficial in terms of improvements in LVEF, blood pressure, heart rate, sympathetic activity, and sleepiness scores in hospitalization and mortality. According to Canadian Continuous Positive Airway Pressure for Patients with Central Sleep Apnea and Heart Failure trial, a physician needs to know whether the apnea is due to OSA or CSA, before prescribing CPAP, as CPAP is not that useful in CSA as in OSA [28]. Later on, in 2013, Noda et al. demonstrated that CPAP effects on both systolic and diastolic BP are related to its compliance [4]. Timely management of OSA with CPAP is required to prevent hypertension and its related complications like CVD and atherosclerosis. This study quoted that CPAP usage reduces LV preload, afterload, and increase in partial pressure of carbon dioxide (PaCO_2_), though the underlying mechanism is not clear [4].

OSA is considered to have an impact on vascular endothelial design and hence is attributed as a sole, additional, or even synergistic risk factor for CVD along with multiple genetic and physical characteristics in both population- and clinic-based studies. Celen et al., by conducting a traditional review, found that CPAP reduced daytime sleepiness, improved quality of life, and in the prognosis of CVD in clinic-based cohorts only [6]. However, many CVD patients with associated OSA do not report daytime sleepiness. Further research is required to define the impact of CPAP as a non-pharmacological intervention for CVD patients with OSA [6]. The table below (Table 2) gives a clear idea about the CV outcomes in sleep apnea patients who were prescribed with CPAP management, whether long-term, short-term or even nasal mode of administration in different studies that we analyzed.

**Table 2:** Major adverse cerebrovascular and cardiovascular endpoints (MACE) with CPAP therapy. CPAP: continuous positive airway pressure; NT-pro BNP: N-terminal pro-brain natriuretic peptide; PVC: premature ventricular contractions; HF: heart failure; OSA: obstructive sleep apnea; CV: cardiovascular; BP: blood pressure

Author/ Year	Purpose of the study	No. of patients	Type of study	Result/ Conclusion
Seyis et al. [1]	Influence of CPAP on ventricular wall stress and PVC in heart failure and OSA patients	80	Observational	NT-pro BNP levels and PVC frequency has reduced with CPAP therapy in HF and OSA patients
Celen et al. [6]	Impact of CPAP on CV events in OSA		Traditional review	Non-pharmacological intervention of CVD and OSA patients with CPAP reduces daytime sleepiness and improves quality of life to some extent
Dong et al. [16]	CPAP in hypertension with CABG and OSA and its effects	59	Observational	With CPAP and standard antihypertensive therapy improvement in non-dipping hypertension and daytime sleep was observed
Kasai et al. [15]	CPAP treatment and prognosis of HF in OSA patients	88	Observational	There is a significant reduction in hospitalization and death rate with CPAP usage
Chowdhury et al. [28]	OSA and CPAP treatment focussed on sleep-disordered breathing and HF		Traditional review	Improvement in LVEF, heart rate, BP, sympathetic activity, hospitalization, and mortality were the advantages of CPAP treatment in OSA
Kaneko et al. [25]	Benefits of CPAP in patients with OSA and HF	24	RCT	SBP reduction and improved LV systolic function is due to CPAP
Noda et al. [4]	Patients with HTN and HF and their therapeutic strategies in sleep apnea		Traditional review	Effective reduction in hypertension and its complications is by management of OSA with CPAP at right time
Kita et al. [8]	Effects of nasal CPAP on nocturnal secretion of cardiac BNP	22	Observational	Reduced BP elevation and BNP and ANP levels with overnight usage of nasal CPAP
Picard et al. [17]	Coexistence of CVD with nocturnal BP fluctuations and OSA treatment with CPAP	86	Observational	Improvement in all polysomnographic parameters in patients on CPAP
Wang et al. [20]	A systematic review and meta-analysis on CV outcomes in CAD and OSA patients with long term CPAP	1430	Systematic review and meta-analysis	Subsequent CV events might be prevented in CAD and OSA patients with the use of CPAP
Labarca et al. [21]	Prevention of cardiovascular events in OSA patients in a systematic review and meta-analysis - efficacy of CPAP	5817	Systematic review and meta-analysis	No sufficient evidence to prove that CV outcomes reduce with CPAP therapy
Abuzaid et al. [22]	CPAP therapy in patients with OSA and CV outcomes - a meta-analysis	3780	Meta-analysis	In moderate to severe OSA patients, the usage of CPAP compared to medical therapy alone doesn't lead to marked decrease in major cardiac events and all-cause cardiac mortality unless CPAP is used for >four hours per night.
Peled et al. [14]	Effects of continuous positive air pressure treatment on nocturnal ischemia in OSA and IHD patients	51	Observational	Improved nocturnal ischemia with CPAP treatment
Glantz et al. [26]	A randomized controlled trial on diastolic function in CAD and non-sleepy OSA patients with CPAP effects	244	RCT	Significant association between good CPAP adherence and an increase in diastolic relaxation velocity after one year.
McEvoy et al. [27]	CV events prevention in OSA patients with CPAP	2717	RCT	Not much difference between CPAP therapy along with usual care and CPAP therapy alone in view of CV events
Campos-Rodriguez et al. [19]	CPAP advantages in CV mortality in women	1116	Observational	Severe OSA and CV deaths are associated and CPAP reduced the risk
Marin et al. [18]	Long-term effects of CPAP in cardiovascular outcomes in men	1651	Observational	Effective nasal CPAP treatment significantly reduces the CV outcome
Patrick et al. [23]	Effect on BP by the CPAP in OSA patients	572	Meta-analysis	Net reduction in mean BP with the usage of CPAP
Hu et al. [24]	Beneficial effects of CPAP in reducing hypertension in sleep apnea patients	691	Meta-analysis	Significant reduction in nocturnal BP was noticed in treatment group compared to control group

Evidence Level

According to our review, all observational studies showed the beneficial side of the CPAP usage in terms of cardiovascular function like improvement in heart failure, hypertension (HTN), post-CABG patients, and CV deaths, while in clinical trials, the usefulness of CPAP was detected only when the patients used it for at least four hours a day for at least one year.

Inflammatory markers

The inflammatory marker, hs CRP, is a serum biomarker of CV injury. A study conducted by Zhao et al. in the year 2011 in Chinese patients with CAD on medications found that hs CRP is independently related to OSA, which shows an association between inflammation and OSA. This elevated hs CRP can be reduced with CPAP management [5]. 

Randomized Intervention with CPAP in Coronary Artery Disease and Sleep Apnea (RICCADSA) trial experimented by Thunström et al. to prove the CPAP effects on inflammatory markers because of the association between non-sleepy OSA and vascular inflammation in CAD patients is evident [29]. The study finally concluded that inflammatory markers didn't change much between the study and control group besides interleukin 6 (IL-6) (it was reduced in both groups irrespective of the treatment) [29]. Many studies showed the effects of CPAP on lipid profile and high sensitive CRP in nonobese patients with OSA and CAD. One among them is an RCT by Huang et al. in 2016 [30]. Seventy-eight nonobese patients on standard lipid-lowering therapy for different reasons showed no difference, but the CPAP management brought the reduction in lipids and hs CRP levels in 12 months duration [30].

Evidence Level

To date, hs CRP is considered a better marker for both inflammation and atherosclerosis together [5]. In our review, the evidence of a reduction in inflammatory markers like hs CRP or IL-6 was equivocal among the observational studies and RCTs.

Limitations

We didn't include animal studies to find the CPAP effects on heart cells. We excluded other language studies except for English. We also couldn't observe whether CPAP is beneficial in pediatric and geriatric groups.

## Conclusions

In this review article, we explored both positive and negative effects of CPAP on the CV system. The impact of CPAP therapy on cardiomyocytes is reflected in the form of changes in cardiac biomarkers like ANP, NT-proBNP (reduced), and highly sensitive troponin C (normal or increased) in OSA patients associated with CVD when compared to patients with OSA alone. While considering the CV outcomes, the results varied based on the type of study conducted. Most of the studies showed that CPAP had positive consequences in terms of improvement in CV events, whereas according to some RCTs and reviews, the results were mostly linked to proper compliance to the CPAP therapy. However, the evidence showing benefits of CPAP usage on inflammatory markers like hs CRP and IL 6 (which serve as a connection between inflammation and atherosclerosis) is inconclusive. Further research is needed to explore the use of CPAP as a sole treatment to reduce the ill effects of sleep apnea on cardiomyocytes.
